# Filament‐Based Melt Electrowriting Enables Dual‐Mode Additive Manufacturing for Multiscale Constructs

**DOI:** 10.1002/smsc.202300021

**Published:** 2023-06-11

**Authors:** Kilian Maria Arthur Mueller, Annika Hangleiter, Sarah Burkhardt, Diana Marcela Rojas-González, Christina Kwade, Sebastian Tobias Pammer, Stefan Leonhardt, Petra Mela

**Affiliations:** ^1^ Chair of Medical Materials and Implants Department of Mechanical Engineering TUM School of Engineering and Design Munich Institute of Biomedical Engineering Technical University of Munich Boltzmannstraße 15 85748 Garching Germany; ^2^ Kumovis GmbH Flößergasse 4 81369 München Germany

**Keywords:** biofabrication, fused filament fabrication, hybrids, melt electrowriting, multiscale

## Abstract

Melt electrowriting (MEW) is an electric‐field‐assisted fiber‐forming biofabrication strategy for the additive manufacturing (AM) of precisely defined 3D microarchitectures. MEW is based on pressure‐driven extrusion of a polymer melt pool, currently mainly implemented at laboratory scale with specialized machine technology and limited to only few materials. This precludes the accessibility of MEW to a broader user group and can become the bottleneck of MEW's technological advancement. In contrast to conventional MEW, a filament‐based approach (F‐MEW) is introduced that exploits the technological ecosystem of fused filament fabrication (FFF), a globally used transformative AM technique. In this work, a polymer filament serves as feedstock material and is melted just on demand. By upgrading existing FFF systems, MEW of polymer microfibers is enabled, as validated with polycaprolactone (PCL) and demonstrated with direct writing of thermosensitive polydioxanone (PDO). Finally, FFF and F‐MEW are hybridized in a dual‐mode AM process. This enables multiscale constructs featuring both FFF struts and one order of magnitude smaller F‐MEW microfibers. This work opens the accessibility of F‐MEW to the large FFF user group, potentially benefitting from the plethora of filaments available for FFF, while, at the same time, expanding the FFF fabrication window.

## Graphical abstract

This work introduces a filament‐based approach to melt electrowriting (F‐MEW) by exploiting the technological ecosystem of fused filament fabrication (FFF) 3D printers. With minimal modifications of existing FFF systems, electrohydrodynamic direct writing of polymer microfibers can be achieved. Converging FFF and F‐MEW enables the fabrication of multiscale constructs with a single print head.

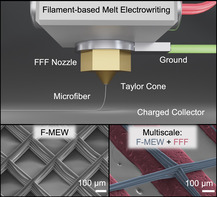

## 1 Introduction

Melt electrowriting (MEW) is an advanced fiber‐forming technique that has convincingly proven its capability to fabricate complex microfiber architectures via additive manufacturing (AM) principles.^[^ ^1^ ^]^ It is part of the wide family of electrohydrodynamic (EHD) processes, that exemplarily have also been applied to dispense liquids on the micro‐ to nanoscale.^[^ ^2^, ^3^ ^]^

In MEW, a polymer melt pool is pneumatically extruded from a feedstock reservoir via a nozzle.^[^ ^4^ ^]^ By applying a high‐voltage electric field between the nozzle and a collector, the extruded polymer melt builds up a Taylor cone at the nozzle tip that transforms into a stable fiber jet.^[^ ^5^ ^]^ While the jet travels toward the collector, it experiences significant thinning,^[^ ^6^ ^]^ resulting in a microfiber with a diameter typically ranging from 2 to 50 μm,^[^ ^7^ ^]^ significantly smaller than diameters typically obtained by other extrusion‐based AM techniques.^[^ ^8^, ^9^ ^]^ Translating the collector with a speed equal or greater than the fiber jet's flight speed enables the deposition of the fiber in a direct writing mode.^[^ ^10^ ^]^ Stacking the deposited fiber along predefined paths results in layer‐by‐layer additively manufactured scaffolds.^[^ ^11^ ^]^ Despite these unique dimensional features and precision, and the resulting wide range of applications,^[^ ^1^ ^]^ MEW is still a niche technique relying mainly on in‐house developed laboratory setups.^[^ ^1^, ^12^, ^13^, ^14^ ^]^

So far, conventional MEW provides the polymer feedstock in a heated plastic or glass syringe over extended periods of time, in the order of hours (for a single print job) and days (repeated use of the polymer in the same syringe),^[^ ^15^ ^]^ which causes significant thermal stress to the melt pool.^[^ ^16^ ^]^ While it has been shown that polycaprolactone (PCL) is robust against this,^[^ ^15^ ^]^ these thermal stresses are detrimental for thermosensitive polymers such as poly(lactic*‐co*‐glycolic acid) (PLGA), which degraded in the melt pool unless a plasticizer was added to lower the melting temperature.^[^ ^16^ ^]^

In contrast to MEW, fused filament fabrication (FFF) is a widely established extrusion‐based AM technique that offers solutions to process thermoplastics on an industry‐compatible technology readiness level,^[^ ^9^, ^17^ ^]^ backed with ever‐expanding technological advancements on both the hardware and software level driven by a broad user base, including academia, industry, and private users.^[^ ^18^, ^19^ ^]^ In FFF, a polymer filament is fed to the print head via a set of pinch wheels and is melted in the nozzle just on demand,^[^ ^20^, ^21^ ^]^ hence obviating the need for a syringe or similar components that provide a melt pool. The resolution of FFF is mainly dictated by the nozzle diameter, which is usually larger than 150 μm, typically around 400 μm,^[^ ^9^ ^]^ and hence at least an order of magnitude larger than that of MEW.^[^ ^7^ ^]^ FFF relies on a wide range of available thermoplastic filaments, from low‐melting degradable polymers^[^ ^22^ ^]^ to high‐performance polymers.^[^ ^23^ ^]^

By enabling an FFF system to perform electrohydrodynamic AM, we envision a filament‐based approach of MEW (F‐MEW) that will leverage established workflows of FFF with the benefit of significantly increasing the accessibility of MEW to a wide user base, and therefore to profit from a continuous innovation push driven by combinatorial efforts of its broad user base, including an expanding choice of filaments. Furthermore, combining F‐MEW with FFF in the same machine brings unprecedented capabilities for multiscale AM, de facto expanding the FFF fabrication window.

## 2 Results and Discussion

### 2.1 Filament‐Based Melt Electrowriting (F‐MEW) Technology

**Figure 1:**
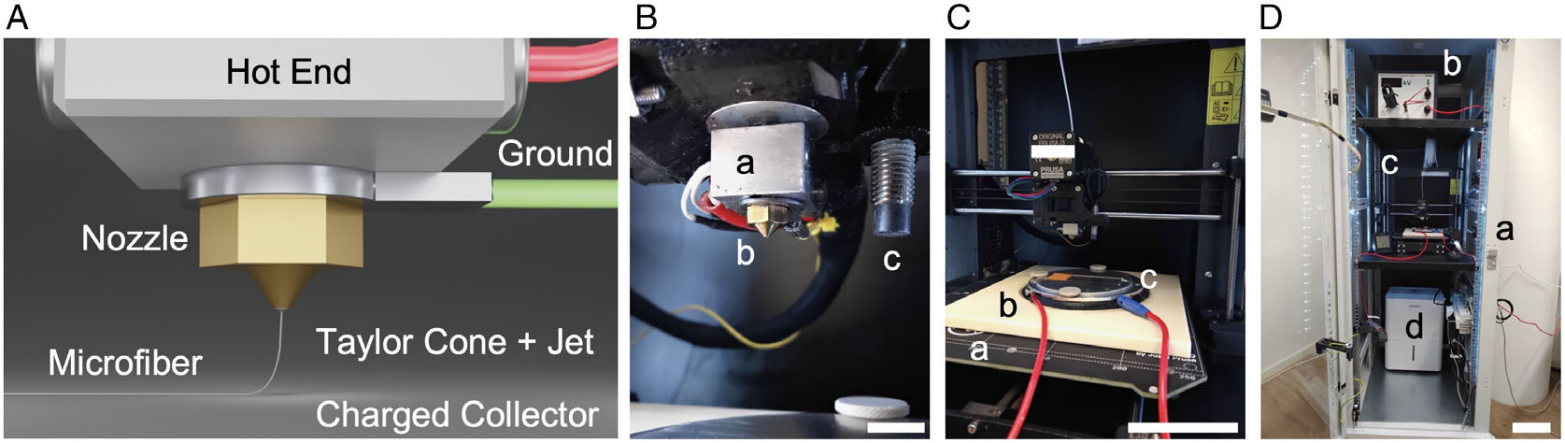
Technology for filament‐based MEW. A) Schematic rendering of the filament‐based electrohydrodynamic writing of a polymer fiber. B) The print head consisted of: a) a commercially available FFF hot end into which b) a standard FFF nozzle was screwed that was electrically ground via a ring cable lug. The print head was leveled to the print bed via c) the printer's PINDA sensor (scale bar: 10 mm). C) The print bed consisted of: a) a standard FFF heated bed onto which b) a ceramic plate was attached. c) The metal collector was fixated on the ceramic plate and connected to a high‐voltage power supply (red cables) (scale bar: 10 cm). D) View into the FFF printer's enclosed build volume. The modified F‐MEW printer was mounted into: a) a server rack that could be closed to shield the user from b) the high voltage applied to c) the F‐MEW system. Inside the rack, the atmosphere humidity was controlled via d) a dehumidifier (scale bar: 20 cm).

We minimally modified a commercially available FFF printer to be able to apply a high‐voltage difference between the head and the collector, as schematically shown in **Figure** **1**A, and thus enable F‐MEW. First, the hot end of the print head with a brass nozzle of 0.25 mm diameter was electrically grounded via a ring cable lug (Figure 1B). Next, the existing metal print bed was advanced to a high‐voltage collector (Figure 1C) by placing a metal sheet on a high‐performance ceramic plate, which electrically insulated the existing metal print bed and the other FFF printer components from the potentially hazardous voltage. Finally, to enable user‐safe F‐MEW, a door‐lock mechanism prevented the operator to interfere with the process when the voltage was activated and automatically decharged the collector when the door was opened (Figure 1D). The kinematics of the FFF system was kept in its original state.

These minimal and low‐cost technological modifications will enable the large FFF community to easily upgrade their existing systems into machines that can perform both FFF and F‐MEW, without the need to purchase a commercially available conventional MEW setup or to custom‐build one.^[^ ^12^, ^13^, ^14^ ^]^

### 2.2 Validation of the F‐MEW Technology

**Figure 2:**
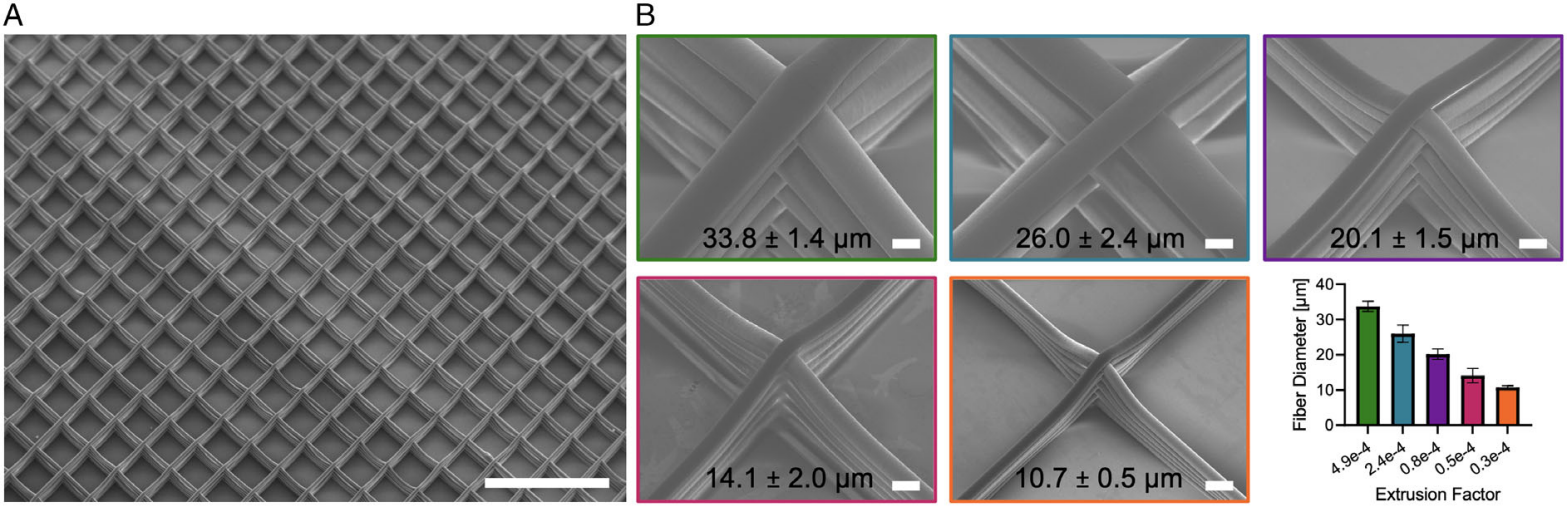
PCL microfibers via F‐MEW. A) PCL microfibers were fabricated in an orderly stacked box architecture (scale bar: 1 mm). B) By varying the pinch wheel‐driven extrusion factor we obtained constant fiber diameters ranging from 33.8 ± 1.4 down to 10.7 ± 0.5 μm (scale bars: 20 μm).

We validated the F‐MEW concept with a PCL filament, as PCL is the current gold standard polymer for MEW.^[^ ^24^ ^]^ The feed rate of the filament into the print head was given as a Gcode command to achieve extrusion. When applying an electric field between nozzle and collector, the extruded melt at the nozzle tip transformed into a Taylor cone from which a fiber jet emerged. While traveling toward the collector, the jet was stabilized by the electric forces, preventing it from breaking up into droplets again caused by Rayleigh–Plateau instabilities,^[^ ^5^, ^25^ ^]^ and experienced significant thinning during its flight phase to well below the diameter of the nozzle orifice, which is characteristic for electrohydrodynamic melt processing.^[^ ^6^ ^]^ Typical MEW layered constructs were obtained (**Figure** **2**A). We tuned the fiber diameter by controlling the extrusion rate as given by the pinch wheel rotation, where an increased number of steps correlated with larger fiber diameters. Specifically, here we demonstrate 10.7 ± 0.5, 14.1 ± 2.0, 20.1 ± 1.5, 26.0 ± 2.4, and 33.8 ± 1.4 μm fiber diameter (Figure 2B). The corresponding extrusion rates are 1.39, 2.19, 2.93, 8.81, and 17.74 μL h^−1^, respectively, and were controlled by the dimensionless extrusion factor, which is here defined as the length of filament (mm) fed into the print head divided by the travelled distance (mm) of the print head within the same time frame. The obtained diameters are in the typical range of conventional MEW^[^ ^1^ ^]^ and the range of fiber diameter can be further expanded depending on the extrusion rate. These fibers were also responsive to the electrostatic autofocusing effect in MEW,^[^ ^26^, ^27^ ^]^ which allowed them to be precisely stacked following the layer‐by‐layer paradigm of AM to form highly ordered structures. Furthermore, F‐MEW exploits on‐demand melt delivery and, therefore, avoids limited feedstock material supply, differently from other electrohydrodynamic processes like pyro‐EHD, which, although able to produce well‐ordered structures, is restricted to the volume of the initial liquid drop.^[^ ^28^, ^29^, ^30^ ^]^

**Figure 3:**
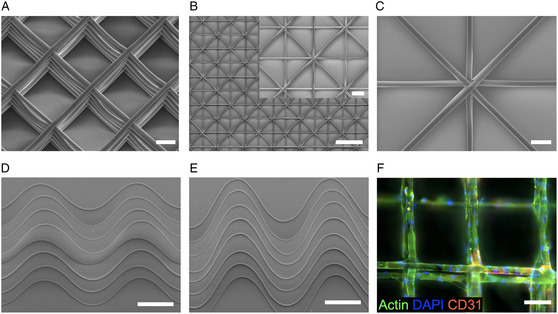
Accurate PCL microfiber deposition via F‐MEW along precoded fiber paths. A) Boxed scaffolds consisting of five layers (scale bar: 100 μm). B,C) Scaffold based on periodically shifted straight lines that result in triangular pores (scale bars: 500 μm: inset: 100 μm). D,E) Sinusoidal microfiber patterns with different amplitudes were printed at CTS (scale bars: 500 μm). F) HUVECs wrapped around PCL microfibers after three days of culture (scale bar: 100 μm).

We highlight the direct writing capabilities of polymer microfibers via the F‐MEW approach by showcasing traditional boxed fiber architectures (**Figure** **3**A) and scaffolds obtained from straight lines with periodical directional shifts as shown in Figure 3B,C. We further highlight the pattern design freedom with exemplary sinusoidal patterns (Figure 3D,E), of which we customized the amplitude. Importantly, these patterns were printed above the critical translation speed (CTS), hence following the sinusoidal Gcode pattern, without fiber coiling artefacts that would occur below the CTS.^[^ ^10^, ^31^ ^]^ However, as direct writing above CTS is inherently linked to a lag in the fiber jet, the as‐printed amplitudes are smaller than the coded ones. Specifically, for prescribed amplitudes of 550 (Figure 3D) and 650 μm (Figure 3E), we measured amplitudes in the printed patterns of 194 ± 23 and 318 ± 24 μm (*n* = 40), respectively. Under ideal print conditions, the fiber jet lag is constant for an optimized parameter set, and, therefore, one can compensate for the loss in the printed amplitudes by oversizing the coded patterns, as previously shown for conventional MEW.^[^ ^27^ ^]^ Boxed scaffolds were cultured with human umbilical vein endothelial cells (HUVECs) and after three days the PCL fibers were supportive of cell adherence and proliferation, as shown by immunostaining of nuclei (blue), actin (green), and CD31 (red) with HUVECs wrapping entirely around F‐MEW microfibers (Figure 3F).

With the aim of reducing the thermal stress to the molten polymer during fiber formation, Luposchainsky et al. recently mounted a FFF head on a robotic arm in order to perform electrohydrodynamic writing of the thermosensitive polymer polydioxanone (PDO) onto a sphere.^[^ ^32^ ^]^ However, direct writing was not achieved because, as the authors explained, the robotic system was not able to consistently move at the CTS in order to deposit the fiber jet in a controlled direct writing mode. This resulted in excessive coiling of the fibers that hence did not reproduce the programmed Gcode and additionally failed to produce orderly stacked scaffolds. Furthermore, using a robotic arm as kinematic system limits the accessibility of MEW instead of capitalizing on the large user group with existing conventional FFF printer kinematics.

**Figure 4:**
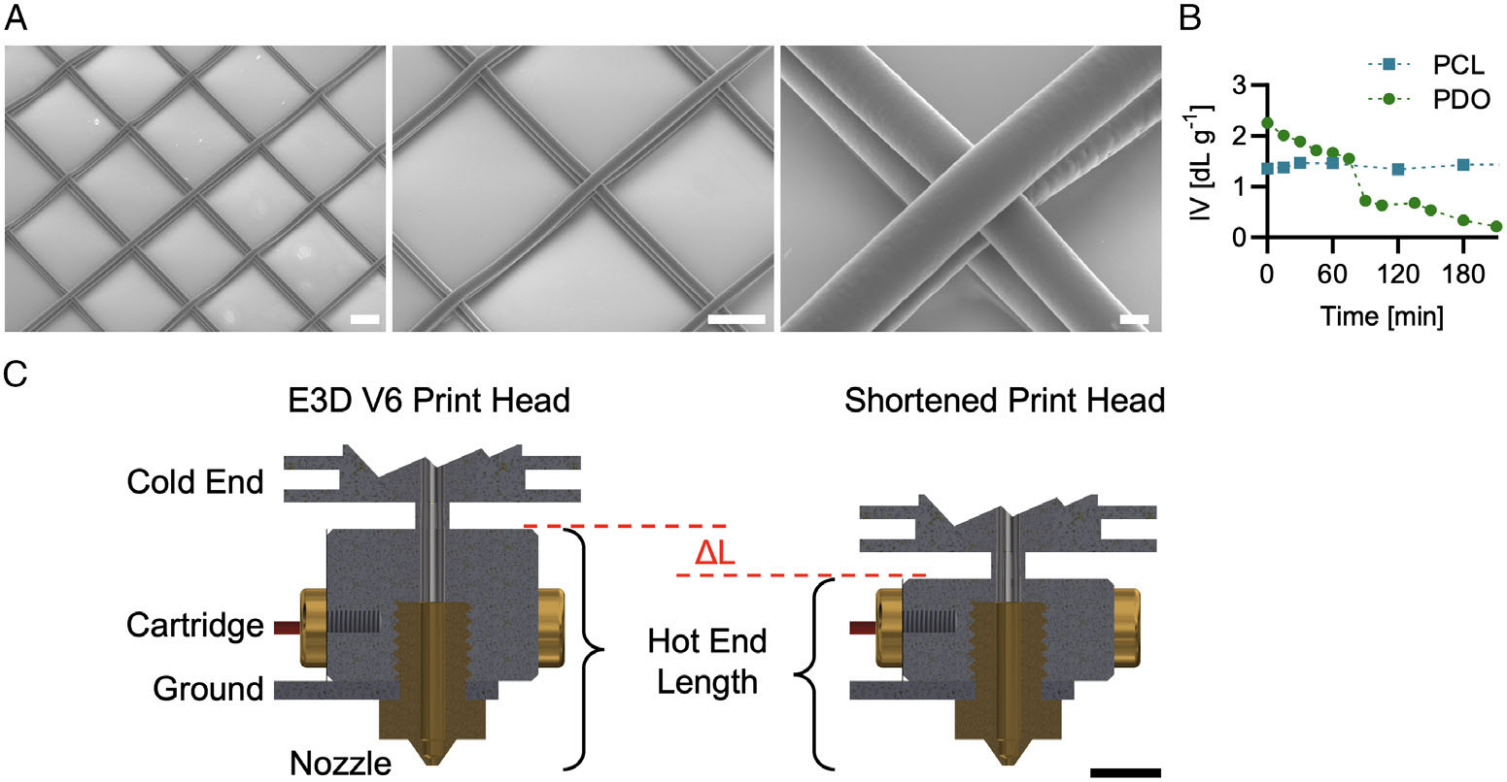
Melt electrowriting of PDO. A) SEM images of PDO scaffolds (scale bars: 100, 100, 10 μm). B) The IV of PDO subjected to 135 °C decreases with time, while it is, in contrast, constant for PCL at 70 °C. C) Cross‐sectional view of a standard E3D V6 print head and the proposed version with a shortened hot end to reduce melt residence time (scale bar: 5 mm).

In contrast, by F‐MEW, we were able to work in the direct writing mode and to fabricate precisely stacked scaffold architectures from PDO microfibers (**Figure** **4**A) as the conventional FFF printer kinematic enabled travel speeds equal and higher than the required CTS. We could, therefore, demonstrate for the first time the MEW of PDO. It is, though, worth noting that the printing conditions would result in a stable jet only for a limited time window, and that to keep producing precise architectures we needed to adjust the parameters. Depending on the nozzle temperature and extrusion rate, this time window was found to be in the range of 30–60 min. The need for parameter adjustments is due to a change in the PDO viscosity due to the degradation of the polymer in the hot end of the print head. It is, indeed, true that the filament is melted on demand in FFF and that, as a consequence, temperature‐sensitive polymers can be processed. However, in the case of MEW, much lower flow rates are needed and, consequently the residence time of the polymer in the hot end is increased significantly. Depending on the printing parameters, this can be in the range of hours. In the case of the fast‐degrading PDO, already one hour of residence time results in a significant drop of the inherent viscosity (IV), as shown in Figure 4B, which makes PDO a particularly challenging material. This is in strong contrast to PCL whose IV at the printing temperature remains constant well beyond the residence time in F‐MEW (Figure 4B). It is, therefore, of critical importance to evaluate the change of material properties at the printing temperature as a function of time to ensure printability by F‐MEW under constant conditions. Other less sensitive materials such as poly(*L*‐lactide) (PLLA)^[^ ^33^ ^]^ can then still benefit from the shorter residence times with respect to conventional MEW.

Nevertheless, in the case of PDO, it was still possible to adapt the printing conditions, for example, by overriding the print speed, to enable direct writing of PDO microfibers over time. In this context, machine vision leveraged self‐correcting systems could be of great benefit as it has been implemented by Mieszczanek et al. in a conventional MEW system.^[^ ^34^ ^]^ However, this comes at the cost of an undefined polymer's state and, therefore, changing properties of the produced patterns. To obviate this problem, we propose to shorten the heating block of the FFF hot end in future works in order to further reduce the inside channel length (Figure 4C). This will potentially result in a melt residence time that is fully compatible with the degradation window of thermosensitive polymers and might propel their accessibility via filaments to the still limited material library of MEW.^[^ ^24^ ^]^ A redesign of the nozzle could also include a coating to tune the interaction with the polymer melt and thus modulate jet formation.^[^ ^35^, ^36^ ^]^

From a technical perspective, it is important to note that in F‐MEW the material feed is discretized by the steps of the extruder motor driving the filament. This leads to pressure pulses in the melt and hence induces variations in fiber diameter and in turn affects the CTS. Therefore, F‐MEW has to be performed with a carefully selected set of parameters to avoid irregularities in the mass flow that would reduce print fidelity. Linear patterns are less sensitive to these more challenging print requirements as they can safely be printed above CTS, as shown in Figure S1, Supporting Information, where a box pattern with mesh size of 300 μm resulted in interfiber distances of 301.2 ± 6.1 and 300.9 ± 4.5 μm in the *X* and *Y* direction (*n* = 32), respectively. Curvilinear patterns can also be achieved, although more challenging and more prone to print defects caused by CTS variations. Nevertheless, we were able to print a curvilinear pattern consisting of semicircles (prescribed radius of 1000 μm) with a resulting radius of 964.8 ± 15.5 μm (*n* = 60, Figure S1, Supporting Information).

Introducing a gear system that reduces the effective motor step size will help minimizing variations in the mass flow. Nonetheless, it is possible to obtain satisfactory fiber patterns via F‐MEW as we have shown.

### 2.3 Dual‐Mode Multiscale AM via Combined FFF and F‐MEW

**Figure 5:**
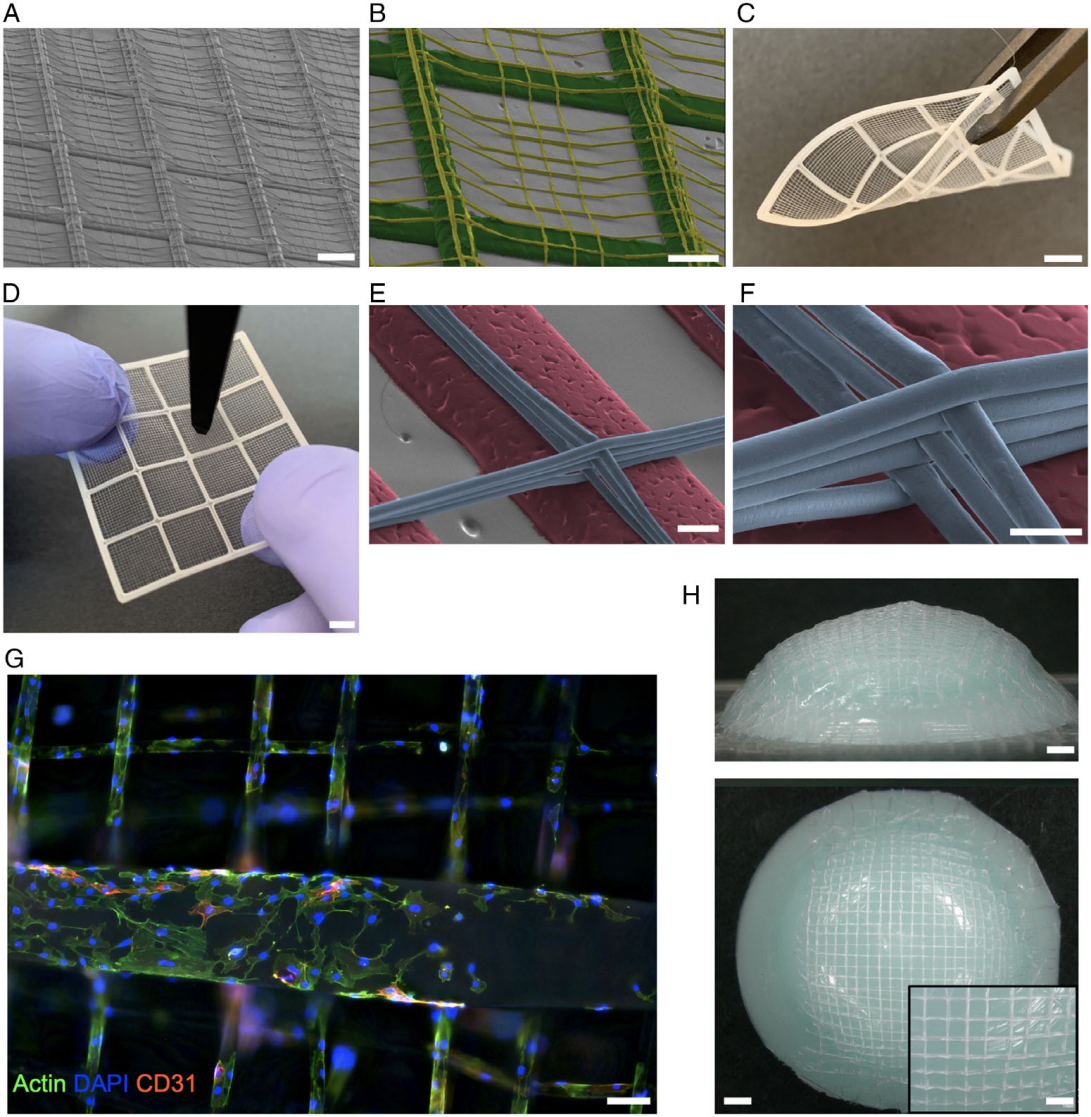
A–H) Dual‐mode multiscale constructs fabricated via a hybrid FFF and F‐MEW process from PCL (A–D,G,H) and PDO (E,F). A) FFF struts were covered with a fine fiber mesh fabricated via F‐MEW (scale bar: 1 mm) as highlighted in (B) with FFF struts colored in green and F‐MEW fibers in yellow (scale bar: 500 μm). C) Such a multiscale construct can easily be handled (scale bar: 5 mm) and D) even withstand the mechanical forces when punctured with tweezers (scale bar: 5 mm). E,F) Dual‐mode multiscale constructs were also fabricated from PDO. FFF struts are shown in red and F‐MEW fibers in blue (scale bars: 100 μm, 50 μm). G) After three days of culture on a multiscale scaffold, HUVECs wrapped around the F‐MEW microfibers while spreading on the surface of the large FFF fibers (scale bar: 100 μm). H) Convex domes were first printed in FFF mode and subsequently covered with a microfiber mesh via F‐MEW to showcase dual‐mode multiscale fabrication of nonplanar components (scale bars: 1 mm, inset: 500 μm). Constructs shown in (A–G) were fabricated on the modified Kumovis printer, while the domes shown in (H) were fabricated on the modified Prusa printer.

As now shown for an affordable desktop FFF system, we, furthermore, similarly upgraded a high‐performance industry‐scale FFF system, that was specifically designed to target the medical device industry by having a cleanroom print chamber. In both cases, while enabling F‐MEW, the FFF mode of the system was still fully functional upon switching off the high voltage and reducing the working distance to 0.2 mm. Hence, multiscale AM could be realized with a single print head by hybridizing the two techniques. This dual‐mode fabrication allowed to combine large FFF structures with microfibrous F‐MEW architectures (**Figure** **5**A), as shown in green (FFF) and yellow (F‐MEW) for a PCL multiscale construct (Figure 5B). This was further exemplary demonstrated for a fine F‐MEW mesh mechanically reinforced with FFF struts for easy handling (Figure 5C). Here, the FFF components served as stringers that supported the F‐MEW mesh even when punctured with tweezers indicating sufficient interfacial bonding (Figure 5D). This was further demonstrated via a delamination test, in which the F‐MEW mesh failed at 1.71 ± 0.52 N, but the F‐MEW/FFF interface remained intact (Figure S2, Supporting Information). The dual‐mode fabrication was also verified for PDO as shown in red (FFF) and blue (F‐MEW) (Figure 5E,F). Similar multiscale constructs were previously reported by exploiting the direct ink writing (DIW) approach of extruding a polymer strand from a syringe container, after switching off the electric field needed for MEW.^[^ ^37^, ^38^ ^]^ However, in DIW the material viscosity is typically limited to the range of 10^2^ to 10^6^ mPa s at a shear rate of 0.1 s^−1^ to ensure printability,^[^ ^8^ ^]^ while the pinch wheel‐driven filament extrusion in FFF polymers is open for a significantly wider viscosity window.^[^ ^39^ ^]^

To showcase the multiscale scaffolds’ relevance for tissue engineering, we also seeded them with HUVECs. Again, after 3 days of culture, the HUVECs wrapped around the F‐MEW microfibers and now additionally spread on the FFF struts (Figure 5G).

Moreover, here we advance hybrid multiscale manufacturing from flat structures to 3D components, as highlighted by patterning a FFF printed dome with a melt‐electrowritten fiber mesh (Figure 5H). This will ultimately empower the microstructuring of anatomically relevant nonplanar surfaces. The dual‐mode concept enabled by F‐MEW closes the fiber diameter gap of combined electrospun (nanometer fibers) and FFF constructs, that typically require two specialized systems,^[^ ^40^ ^]^ by offering now an intermediate fiber diameter range (single to double digit micrometer) via F‐MEW for hybrid FFF composite constructs—all from a single print head.

## 3 Conclusion

This work presents a filament‐based approach to MEW by exploiting the FFF technology ecosystem. F‐MEW takes advantage of the melt‐on‐demand concept offered by the pinch wheel‐driven filament extrusion known from FFF. Electrohydrodynamic fiber formation from PCL and PDO melts was achieved at voltages of −4.5 to −5.5 kV applied to the print bed while the commercial FFF nozzle (0.25 mm diameter) was electrically grounded. PCL microfibers with a tunable diameter ranging from 33.8 ± 1.4 to 10.7 ± 0.5 μm were accurately deposited to form layered scaffolds.

The F‐MEW concept holds great potential to introduce MEW to a much wider user group and can now be easily implemented into existing FFF machines. The compatibility of the two techniques FFF and F‐MEW ultimately enables their convergence and significantly widens the fabrication window by empowering hybrid multiscale AM with a single print head.

## 4 Experimental Section

4.1

4.1.1

##### Polymer Filaments

The polycaprolactone (RESOMER Filament C D1.75) and polydioxanone (RESOMER Filament X D1.75) technical grade filaments had a diameter of 1.75 mm and were used as received from Evonik (Evonik Industries AG, Germany).

##### F‐MEW System

A commercially available FFF system (Prusa i3 MK3s+, Czech Republic) was modified to enable the F‐MEW mode, by adapting the print head and the print bed to implement an electrical field. The print bed consisted of a high‐performance ceramic (DEGUSSIT AL23, KYOCERA Fineceramics Precision, Germany) plate onto which a metal sheet was attached that was connected to a high‐voltage supply (PHYWE, Germany). The ceramic insulated the print bed when a high voltage was applied to the custom‐made two‐part aluminum collector. A double pinch wheel extruder (Bondtech, Sweden) fed the filament to a standard FFF print head (V6, E3D, UK) with a brass nozzle diameter of 0.25 mm. The nozzle was electrically grounded via a ring cable lug that was fixed between the hot end and the nozzle. In this way, we were able to safely generate the high‐voltage electric field between the nozzle and the modified collector required for electrohydrodynamic processing of polymer melts. The upper part of the collector was designed to reproducibly host a microscopy slide (VWR, Belgium). This aluminum holder was screwed to the base of the collector with three polyetheretherketone (PEEK) screws. In addition, the collector base, which was connected to the high‐voltage supply, was bonded to the ceramic insulator with a high‐performance silicone sealant (Makra Norbert Kraft, Germany) to prevent displacement during the printing process.

Additionally, this upgrade methodology for F‐MEW was equally implemented into a commercially available high‐performance FFF‐system (HTRD1, Kumovis, Germany).

##### F‐MEW Printer Control

All factory settings of the printer's firmware were retained, with exception for the minimal temperature to allow extrusion starting from 45 °C for PCL, which melted at 60 °C. Next, we defined the extrusion quantity as the length of forward‐driven filament in relation to the traveled printing distance to be extruded in regular intervals. The network‐based software Repetier‐Server Pro 1.4.2 (Hot‐World, Germany) running on a Raspberry Pi 4 (Model B+, Raspberry Pi, UK) allowed direct transfer of Gcode to the printer, visual observation of the process, and predictive intervention in the process by overwriting or inserting commands into the running program code.

##### Gcode Generation

Gcodes were generated using a custom‐written spreadsheet program (Excel 2019, version 1808, Microsoft Cooperation, United States of America). This program not only specified the coordinates for movement control for the kinematic system (via G1 commands), but also coordinated the extrusion of filament (via E commands) in correspondence with the travelled distance. In this way the extrusion was coupled and discretized to movement commands understandable for a standard FFF printer. To this end, we defined an extrusion factor as the length of filament (mm) fed into the print head divided by the travelled distance (mm) of the print head within the same time frame. This resulted in a dimensionless factor that controlled the material extrusion rate.

##### Process Parameters

All experiments were performed at room temperature and relative humidity between 25% and 35%. For F‐MEW, the nozzle‐to‐collector distance was set to 2.6 mm and electrohydrodynamic processing of polymer melts (PCL at 70 °C/PDO at 115 °C to 140 °C) was enabled with a voltage of −4.5 kV for all PCL experiments, while for PDO voltages between −5.5 and −3.5 kV were applied. Further process parameters for PCL and PDO in F‐MEW and FFF modes are listed in **Table** **1**.

**Table 1:** Parameters for the processing of PCL and PDO in both modes F‐MEW and FFF. FFF Gcodes were generated with the slicing software Simplify3D (Simplify3D, USA)

Filament	Mode	Print head temp. [°C]	Relative speed [mm min^−1^]	Extrusion factor [‐]	Print bed temperature [°C]
PCL	F‐MEW	70	200–320	3.1 × 10^−5^ to 4.9 × 10^−4^	30
	FFF	70	700	–	30
PDO	F‐MEW	115‐140	240–450	3.8 × 10^−5^ to 7.3 × 10^−4^	70
	FFF	130	700	–	70

##### Scanning Electron Microscopy

Samples were stored overnight in a desiccator before being sputtered coated with a 7 nm gold layer and visualized with a JSM‐6390 (Jeol, Germany, accelerating voltage 10 kV). Fiber diameters (*n* = 4) were measured with the scanning electron microscope's built‐in software. SEM images were colorized using Mountains 9 (Digital Surf, France).

##### Inherent Viscosity

PDO and PCL filament samples were subjected to a heat treatment (135 °C for PDO, 70 °C for PCL) in a drying oven (9110‐0082, Binder, Germany) to simulate the thermal load by F‐MEW for up to 210 min.

At the respective time points, filament samples (*n* = 3) were taken out of the oven and cooled down to room temperature to measure their IV. PDO samples were dissolved in hexafluorisopropanol (HFIP, 1 mg PDO in 1 mL HFIP) and stirred for 20 h using a magnetic stirrer before measuring the IV with a rolling‐ball viscometer (Lovis 2000 M/ME, Anton Paar, Austria) using the Lovis standard method with a Lovis angle = 70°, *T* = 30 °C, and Lovis density = 1.5960 g cm^−3^. PCL samples were dissolved in chloroform (1 mg PCL in 1 mL chloroform) and magnetically stirred for 20 h. The IV was measured with the following parameters: Lovis angle = 19°, *T* = 25 °C, and Lovis density = 1.4800 g cm^−3^.

##### Cell Seeding on Scaffolds

The scaffolds (10 mm × 10 mm) were sterilized using 70% ethanol and then washed 3 times in phosphate‐buffered saline (PBS, Sigma, Germany). HUVECs (Promocell, Germany) were seeded directly onto the scaffolds that were placed in 48 well‐plates (Greiner, Germany). A total of 300.000 cells were resuspended in 100 μl of culture medium, pipetted onto the scaffold surface, and incubated for 1 h at 37 °C to allow for cell adhesion. Subsequently, additional 500 μl of endothelial cell basal medium (EBM‐2, Lonza, Switzerland) supplemented with the endothelial growth medium 2 kit (EGM‐2 supplement kit, Lonza, Switzerland) were added to the wells. The cell‐seeded scaffolds were kept in an incubator at standard culture conditions (37 °C, 5% CO_2_) for 3 days.

##### Immunofluorescence

Samples were fixed for 1 h using methanol‐free 4% formaldehyde (Roth, Germany) prepared in PBS followed by a washing step in PBS. Fixed samples were permeabilized using 0.1% Triton x‐100 (Sigma, Germany) and treated with 5% normal goat serum (NGS, Dako, USA) for 1 h at room temperature. They were then incubated with 100 nm working solution of fluorescent phalloidin (Acti‐Stain 488, Cytoskeleton, USA) for 1 h at 37 °C, followed by a washing step with PBS and incubation for 1 h at 37 °C with primary antibody mouse monoclonal anti‐CD31 (1:100, Sigma, Germany). They were then washed with PBS and incubated for 1 h at room temperature with Alexa 594‐conjugated secondary antibody (1:400, Life Technologies, USA). Finally, nuclei were counterstained with DAPI (Carl Roth, Germany). Visualization was carried out using a fluorescence microscope (BZ‐X800, Keyence, Germany).

##### Delamination Test

To quantify the interface quality between the F‐MEW fiber mesh and FFF struts in multiscale scaffolds, we performed a delamination test (*n* = 11) using a Zwickline Z2.5 (ZwickRoell, Germany) tensile tester equipped with a 10 N load cell at a cross head speed of 100 mm min^−1^. A pin of 8 mm diameter was attached to the moving crosshead to puncture the F‐MEW fiber mesh between a square of 10 mm × 10 mm formed by the FFF struts while the construct was locally fixed. During the displacement of the pin, the maximum force before construct failure was recorded.

##### Statistical Analysis

Data are reported and visualized as mean ± standard deviation.

## Conflict of Interest

S.L. and S.P. are employed at Kumovis GmbH. The other authors declare no conflict of interests.

## Supporting information

Supplementary Material

## Data Availability

The data that support the findings of this study are available from the corresponding author upon reasonable request.
