# Supplementary material for: Filament‐Based Melt Electrowriting Enables Dual‐Mode Additive Manufacturing for Multiscale Constructs
Source: Small Sci. 2023 Jun 11;3(8):2300021. doi: 10.1002/smsc.202300021 (PMC11935881; doi:10.1002/smsc.202300021)
Supplement: Supplementary file 1 — Supplementary Material [file SMSC-3-2300021-s001.pdf]

Supporting Information

Filament-Based Melt Electrowriting Enables Dual Mode Additive Manufacturing for Multiscale Constructs

*Kilian Maria Arthur Mueller[1], Annika Hangleiter[1], Sarah Burkhardt, Diana Rojas-González, Christina Kwade, Sebastian Tobias Pammer, Stefan Leonhardt, Petra Mela**

[1] These authors contributed equally to this work

**Corresponding author, petra.mela@tum.de*

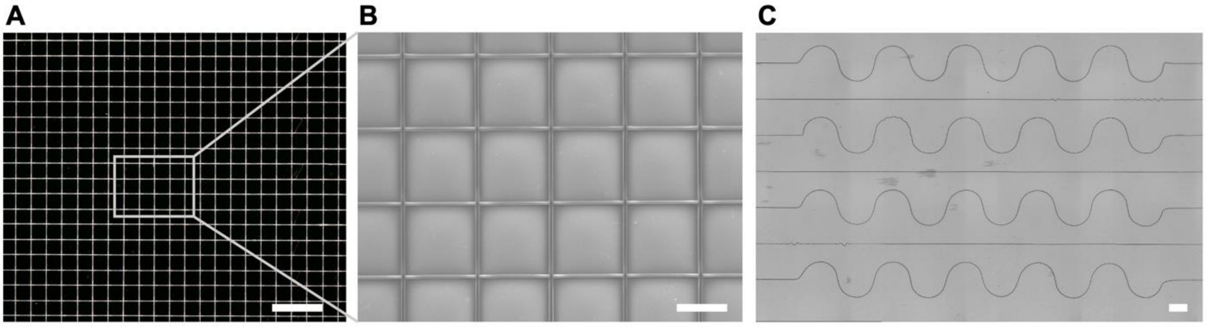

Figure S1: Print fidelity evaluation. A) For a box-pore scaffold with a coded pore size of 300 µm we measured 301.2 ± 6.1 µm and 300.9 ± 4.5 µm interfiber distance in X and Y direction, respectively, from light microscopy images (Keyence VHX-7000, Jeol JSM-6390) using the microscopes' built-in software (scale bar 1000 µm). B) Magnified view of the same scaffold via scanning electron microscopy highlighting the accurate fiber placement (scale bar 200 µm). C) Curvilinear patterns with a prescribed radius of 1000 µm resulted in printed radii of 964.8 ± 15.5 µm (scale bar 1000 µm).

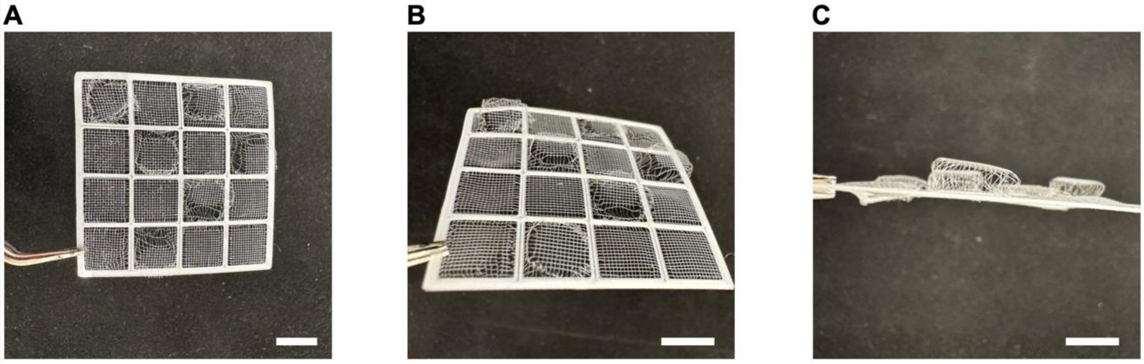

Figure S2: Multiscale scaffolds after the delamination test. A) An 8 mm diameter pin was used to puncture the F-MEW mesh between a square of 10 mm x 10 mm formed by the FFF struts. B) Plastic deformation of the F-MEW mesh caused construct failure while C) the F-MEW/FFF interface remained intact, and the strained fibers were still bonded to the FFF struts (scale bars 10 mm).
